# Measles vaccination coverage, determinants of delayed vaccination and reasons for non-vaccination among children aged 24–35 months in Zhejiang province, China

**DOI:** 10.1186/s12889-018-6226-7

**Published:** 2018-11-27

**Authors:** Yu Hu, Ying Wang, Yaping Chen, Hui Liang, Zhiping Chen

**Affiliations:** Institute of Immunization and Prevention, Zhejiang Center for Disease Control and Prevention, No. 3399 Binsheng Road, Binjiang District, Hangzhou, People’s Republic of China

**Keywords:** Measles, Vaccination, Coverage, Determinants, Children

## Abstract

**Background:**

This study was aimed to assess the coverage of two doses of measles vaccine and identify the determinants of the delayed vaccination.

**Methods:**

A cluster survey among 1386 children aged 24–35 months was conducted. Characteristics on demographic and socio-economic and vaccination records was collected. The overall coverage was defined as the proportion of children receiving the first dose of measles vaccination and the second dose of measles vaccination by 24 months of age. The age-appropriate coverage was defined as the proportion of children receiving the measles vaccine doses within one month after its relevant due date. Timeliness was evaluated with the Kaplan-Meier analysis. Cox proportional hazard regression was adopted to identify determinants of the delayed vaccination.

**Results:**

The overall coverage was 96.9% for the first dose of measles vaccine and 93.9% for the second dose of measles vaccine. The age-appropriate coverage of the first and the second dose of measles vaccine was 76.6 and 68.2%, respectively. Household having more than one child, non-local children were associated with the delayed vaccination for the first and the second dose of measles vaccine. Children delivered at home, younger mothers, low maternal education background, mothers with a fixed job, and low household income were associated with the delayed vaccination for the second dose of measles vaccine.

**Conclusions:**

The coverage of measles vaccine had been improved for both the first and the second dose, while the timeliness still needed improvement. We suggested the policy-makers pay more attention to the reasons for non-vaccination and determinants of delayed vaccination when planning efforts to ensure the high age-appropriate coverage of measles vaccination.

## Background

Measles is a common infectious disease and is considered as a significant cause of child’s mortality, with more than one million measles deaths reported annually worldwide [1]. Measles can be stopped by vaccination with measles-containing vaccine (MCV), which is one of the most cost-effective public health interventions. Due to the highly contagious nature of measles [2], the World Health Organization (WHO) suggests that over 95% of the target population should be immunized with two doses of MCV to halt measles transmission [1]. Missed individuals, vaccinated individuals with primary vaccination failure or immunity waning are susceptible. Furthermore, the secondary attack rate is around 90% among the susceptible population [1]. As such, the second dose of MCV is critical in resolving the primary vaccination failure or boosting the antibody titers after the immunity waning.

Apart from obtaining the vaccination coverage, timeliness is another important index for the success of control and elimination of measles. It is necessary to get measles vaccinations at the recommended ages which are set based on balance between the risk of infection and the immunogenicity of MCV. Delayed vaccination enlarges the gap between the protection from vaccination and the loss of maternal antibodies, which weakens the herd immunity and increases the risk of outbreaks or epidemics. Vaccination coverage is an indicator widely applied for evaluating the performance of expanded program on immunization (EPI), but in these estimates it is difficult to assess the timeliness as it excludes the precise age of administration.

As the measles elimination activity requires an extremely high level of coverage, planning and implementing an effective measles vaccination program is critical. Since 1980s, the Chinese expert advisory committee on immunization program recommended a two-dose MCV vaccination schedule for all children in routine immunization [3]. In 2004, the schedule of MCV was updated, with the first dose of MCV (MCV1) at 8 months of age and the second dose of MCV (MCV2) at 18 months of age, with a minimum interval of 28 days between two doses. MCV vaccinations are included in the Chinese EPI and provide to all children free of charge.

Though the Chinese government had initiated the measles elimination activity since the beginning of this century as a part of the global vaccine action initiative, the outbreaks of measles occurred periodically in every 2–3 years. In Zhejiang province, the measles incidence rates of 2005 and 2008 were both over 30 per 100,000, which were three times as the provincial average incidence of the last two decades. Outbreaks resulted in 14,317 cases in 2005 and 12,782 cases in 2008, and mostly impacted children under three years of age [4]. According to the estimates of vaccination coverage of MCV among children born from 2005 to 2011 based on Zhejiang provincial immunization information system (ZJIIS), the coverage of MCV1 was 98.6% while the coverage of MCV2 was 82.8% [5], much lower than the threshold of coverage of MCV vaccination for maintaining the herd immunity. Additionally, this study only included the children registered in ZJIIS, which would inflate the vaccination coverage due to a common perception that children not registered were also likely to miss the vaccinations. Another coverage survey which was conducted in 2011 in 18 counties of Zhejiang province showed that the coverage of MCV1 was 92.7% but its timeliness was only 59.3% [6]. The lower coverage of MCV and the poor timeliness were considered as the “significant off-track” to meet measles elimination goals.

In order to improve the coverage of complete two doses of MCV vaccination and its timeliness, Zhejiang provincial center of disease control and prevention (CDC) encouraged all immunization providers to adjust the frequency of vaccination service, promote the health education and social mobilization, and launch a training program towards vaccinators to emphasize the importance of MCV vaccinations. However, we had not systematically evaluated the effects of these initiatives at the provincial level. As such, the 2016 Zhejiang provincial coverage survey was conducted among children aged between 24 and 35 months of age to evaluate (1) whether there was a progress in the coverage rates of MCV since 2011; (2) the timeliness of MCV vaccinations; (3) the reasons for non-vaccination of MCV; and (4) the potential determinants for delayed MCV vaccinations.

## Methods

### Study design and setting

Our study was a cross-sectional survey on vaccination coverage among children 24–35 months of age at Zhejiang provincial level. Zhejiang province locates on the east coast line of China and consists of 11 cities, 90 counties, and 1319 towns, with a geographic area of 101,800 km^2^. Zhejiang province, which is one of the densely populated provinces in China, has a population of 72 million (2016 census). Zhejiang province had annual population growth rate of 12.5% and had an estimated 726 thousands births in 2016. Zhejiang province.

### Study population and sampling procedure

Children born from September 1, 2013 to July 31, 2014 (24–35 months of age) were investigated through a household-based cluster survey in August, 2016. The main reason for choosing this age range was that all target children had the chance to finish the full series of MCV vaccination.

The procedure of survey included four steps: first, we applied the probability proportional to population size method to select six towns for every city, with the population size of each town from the census data 2010. Second, we drew lots to choose one community from the selected town. Third, we selected the index household from the selected community using the random number table. Fourth, we visited the adjacent household on the right of the index household when we finished the previous investigation. The remaining 20 households were investigated in the same way. If there were more than one eligible child in household, we drew lots to investigate only one. Households where somebody lived but with no response should be re-scheduled for another attempt. If we did not find enough eligible children in the selected community, the closest community were chosen to investigate the remaining children.

### Sample size

The immunization cluster survey recommended by WHO was adopted in this study. The sample size was calculated based on the following formula [7]: ${\mathrm{N}}_{\mathrm{min}}= deff\times \frac{z_{\left(1-\alpha /2\right)}^2\times p\times \left(1-p\right)}{d^2}$. A two-tailed *α* error of 5%, a permissible error (*d*) of 0.1 on the completeness of two-dose MCV, an expected full coverage of the two-dose of MCV (*p*) of 80% and a design effect (*deff*) of 2 were used to estimate the coverage at city level. The minimum sample size for each city was 123. Finally, the sample size was 126 of each city for the convenience of field survey, which was divided into 21 children in each of the six cluster (town). The sample size for the entire province was 1386.

### Data collection

Parents of the eligible children were surveyed by the vaccination staff from 11 CDCs at city level, who attended a half-day training before the investigation. The training focused on the objectives of the survey, the meaning of every item of the questionnaire, and the skill of investigating the sensitive items. These vaccination staff were not responsible for the investigation in the particular area where they normally worked to avoid the selection bias.

Characteristics on the socio-economic and demographic of the surveyed child, his/her mother and household were gathered by a pre-tested questionnaire developed by Zhejiang CDC. Vaccination status were transcribed from the immunization cards and validated through ZJIIS as the illegible handwriting on some immunization cards. Only written vaccination records were included in the analyses. Any child without written evidence of receiving vaccinations from immunization cards kept by parents or ZJIIS was considered as non-vaccination. The reasons for non-vaccination for each MCV vaccination were investigated with the two questions as following: “why did your child not receive MCV1?” and “why did your child not receive MCV2?” The investigators would read out all eight potential choices of each question and the most likely response was chosen by the surveyed parents.

### Measurements

Two kinds of vaccination coverage was defined in this study. First, the overall coverage for each dose of MCV was defined as the proportion of the surveyed children receiving MCV1 or MCV2 by 24 months of age, regardless of the actual age at vaccination. Second, the age-appropriate coverage was defined as the proportion of the surveyed children receiving MCV vaccinations complying with the schedule, with receiving MCV1 in 8 months of age (between 244 and 275 days of age) or MCV2 in 18 months of age (between 549 and 580 days of age). We considered a month to be 30.5 days on average in this analyses. The assumption for this definition was based on the ideal status that all the vaccinations should be administrated within one month after its relevant due date.

### Statistical analysis

STATA 11 (Stata Corp. 2009, Stata statistical software, college station, TX, USA) were adopted for all data analyses. We used the svy function in STATA 11 to take into account for the cluster into the analyses. The analyses procedures included three steps: First, the characteristics on the demographic and socio-economic were described, using the proportion for categorical variables or the mean for continuous variables. Second, the coverage rate with its 95% confidence interval (*CI*) was calculated by city according to the two different definitions mentioned above. Third, the timeliness of MCV1 and MCV2 was estimated at age *t*, by using the inverse Kaplan-Meier survival function, or 1-SKM(*t*) [8]. Children missing the MCV1 or MCV2 by 24 months of age were defined as censored. Fourth, the proportions of reasons for non-vaccination were calculated and added up. Fifth, the Cox proportional hazard regression model was adopted to explore the determinants of the delayed MCV1 and MCV2, by setting date of birth as the origin time and date of the survey as the exit time. All variable on the demographic and socio-economic were included in the model. The final model was fitted by the backward selection with a significance level of 0.05. The hazard ratio (HR) indicated the likelihood of a child to be administrated timely was presented.

## Results

### Characteristics on demographic and socio-economic

We approached 1422 eligible children to recruit 1386(97.5%) children and their parents who agreed to participate in the survey. Table 1 presented the characteristics on demographic and socio-economic of the surveyed children.

**Table 1:** Summary distribution of the demographic and socio-economic characteristics of children aged 24–35 months, in Zhejiang province

Variables	No. of children (%)
Sex	
Male	700 (50.5)
Female	686 (49.5)
Number of siblings	
1	908 (65.5)
2	362 (26.1)
≥ 3	116 (8.4)
Place of delivery	
Hospital	1285 (92.7)
Home	101 (7.3)
Age of mother (years)	
< 30	881 (63.6)
≥ 30	505 (36.4)
Maternal education level	
< senior middle school	274 (19.8)
≥ senior middle school	1112 (80.2)
Maternal employment status	
Home fulltime	315 (22.7)
Employed	1071 (77.3)
Residence	
Urban	710 (51.2)
Rural	676 (48.8)
Immigration status	
Resident	843 (60.8)
Non-local	543 (39.2)
Family income per month (CNY) ^a^	7336.9 ± 82.3

^a^: continuous variable, presented as Mean ± S.D

### Coverage and timeliness

**Fig. 1:**
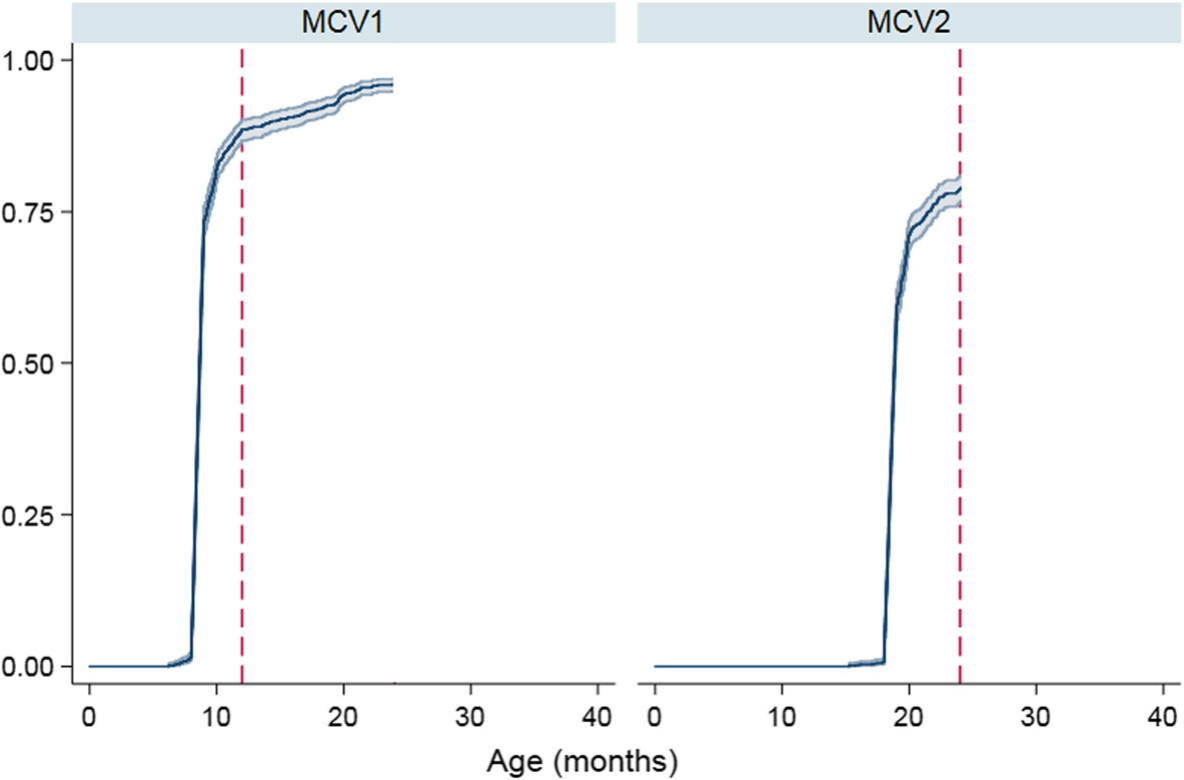
Cumulative vaccination coverage of MCV1 and MCV2 among children 24–35 months of age, in Zhejiang province

Table 2 presented the coverage of MCV1 and MCV2 based on the two different definitions of vaccination coverage. The overall coverage was 96.9% for MCV1 and 93.9% for MCV2 at provincial level. The age-appropriate coverage was 76.6% for MCV1 and 68.2% for MCV2 at provincial level. The coverage of timeliness by 24 months of age was 88.9% for MCV1 and 80.7% for MCV2 at provincial level (Fig. 1).

**Table 2:** The overall coverage and the age-appropriate coverage of MCV both by 24 months of age among children aged 24–35 months in Zhejiang province (total sample = 1386, 126 for each city)

City	Overall coverage (n, %)		Age-appropriate coverage (n, %)^a^	
	MCV1	MCV2	MCV1	MCV2
Hangzhou	125 (99.2)	125 (99.2)	102 (81.0)	93 (73.8)
Ningbo	124 (98.4)	117 (92.9)	97 (77.0)	90 (71.4)
Wenzhou	111 (88.1)	106 (84.1)	88 (69.8)	79 (62.7)
Jiaxing	125 (99.2)	119 (94.4)	100 (79.4)	91 (72.2)
Huzhou	124 (98.4)	118 (93.7)	99 (78.6)	87 (69.0)
Shaoxing	122 (96.8)	118 (93.7)	96 (76.2)	85 (67.5)
Jinhua	123 (97.6)	117 (92.9)	96 (76.2)	82 (65.1)
Quzhou	122 (96.8)	118 (93.7)	95 (75.4)	84 (66.7)
Zhoushan	122 (96.8)	118 (93.7)	99 (78.6)	84 (66.7)
Lishui	115 (91.3)	113 (89.7)	92 (73.0)	82 (65.1)
Taizhou	120 (95.2)	117 (92.9)	97 (77.0)	88 (69.8)
Total	1343 (96.9)	1285 (93.9)	1061 (76.6)	945 (68.2)

^a^: one month after its due date

### Reasons for non-vaccination

Children being sick when the MCV vaccinations were due (27.0%) was the most frequent reason for non-vaccination, followed by the immunization clinic was overcrowded (25.0%), and the schedule of the immunization clinic was conflicted with the working hours (15.9%) (Table 3).

**Table 3:** Reasons for non-vaccination among children aged 24–36 months, in Zhejiang province (total sample = 1386, 3.1% for non-vaccination of MCV1 and 7.3% for non-vaccination of MCV2)

Reasons	MCV1(*N* = 43)	MCV2(*N* = 101)	All(*N* = 144)
Without written vaccination records	4 (9.3)	4 (4.0)	8 (5.6)
Was not aware that the child needed this vaccination	2 (4.6)	7 (6.9)	9 (6.3)
The immunization clinic was overcrowded	12 (27.9)	24 (23.8)	36 (25.0)
The schedule of the immunization clinic was incompatible with working hours	7 (16.3)	16 (15.8)	23 (15.9)
Fear of adverse events	–	5 (5.0)	5 (3.4)
The child was sick when the vaccination was due	10 (23.3)	29 (28.7)	39 (27.0)
The physicians had contraindicated the vaccine	8 (18.6)	11 (10.9)	19 (13.2)
Considered vaccination not important	–	5 (4.9)	5 (3.5)

### Determinants related with delayed MCV vaccination

Household having more than one child, non-local children were associated with delayed vaccination of both MCV1 and MCV2. Except for these two determinants, Children delivered at home, younger mothers, low maternal education background, mothers with a fixed job, and low household income were associated with delayed vaccination of MCV2 (Table 4).

**Table 4:** Hazard ratio (with 95% *CI*) for delayed vaccination of MCV1 and MCV2 among children aged 24–35 months, in Zhejiang province (Cox proportional hazard regression, *N* = 1386) ^a^

Demographic and socio-economic variables		MCV1	MCV2
Number of siblings	1	1	1
	2	1.2 (0.9–1.8)	1.1 (0.9–1.5)
	≥3	**1.6 (1.1–2.0)** ^*^	**2.3 (1.6–3.5)** ^**^
Place of delivery	Hospital	1	1
	Home	1.1 (0.8–1.7)	**1.7 (1.3–2.8)** ^*^
Age of mothers (years)	< 30	1	1
	≥30	0.9 (0.7–1.24)	**0.8 (0.6–0.9)** ^*^
Mother’s education level	< senior middle school	1	1
	≥ senior middle school	0.8 (0.6–1.2)	**0.7 (0.5–0.9)** ^*^
Maternal employment status	Home fulltime	1	1
	Employed	1.2 (0.8–1.5)	**2.3 (1.8–4.0)** ^**^
Immigration status	Resident	1	1
	Non-local	**2.6 (1.9–4.7)** ^*^	**2.9 (2.2–5.3)** ^**^
Family income per month ^b^		0.9 (0.8–1.0)	**0.8 (0.7–0.9)** ^*^

^a^: two variables (sex and residence) were excluded from the backwards model selection

^b^: continuous variable, increasing direction. HR presented in bold were significant with ^*^p < 0.05; ^**^p < 0.01

## Discussion

For the whole province, the coverage of MCV1 reached the goal of 95% set by WHO while the coverage of MCV2 was still below the threshold for stopping the measles transmission. We found that there was 3% children drop out the MCV2 after receiving the MCV1. Our study also found the disparities among cities which might be masked by the provincial estimate. For example, the coverage rates of MCV1 and MCV2 in Wenzhou were all below 90%, which would put children at the risk of measles infection or outbreak. The inequalities of vaccination coverage had been reported in a previous study from the low- and middle- income counties due to the unbalanced socio-economic development [9].

Our previous studies [4, 7] had already emphasized the importance of measuring timely vaccination since simply focusing the coverage at a given age would overrate the real protection in population. The significance of our point was that the sub-population with the delayed vaccination were posed at the unnecessary risk of diseases. Consequently, outbreaks would occur if the epidemic threshold was surpassed and it might spread much faster when the delayed vaccination was coupled with a low vaccination coverage. Compared with the provincial estimates of a similar survey in 2011, the timeliness of MCV1 vaccination had been improved significantly (76.6% in 2016 vs.59.3% in 2011 survey). Actually, it was prioritized to immune children at the appropriate ages and reduce delays after the 2011 survey. CDCs at province and city levels made great efforts to enhance timeliness, mainly through raising awareness of providers and sending reminders to parents. Timeliness had also been considered as an important indicator for assessing the performance of the local immunization program since 2011, in additional to the traditional indicator like the vaccination coverage. Despite of the encouraging improvement, significant gaps still existed. The coverage threshold of 95% was considered to confer measles herd immunity. However, the timeliness of MCV1 and MCV2 still below 90% in this study. The suboptimal timeliness extended the risk time of measles infection for children. Timeliness of MCV vaccination reported in other settings varied, for example, the delayed proportions ranged from 19 to 78% for MCV vaccination in other developing countries [3, 10, 11]. The delayed MCV vaccination was also observed in Switzerland [12], where children spent on average 266 days susceptible to measles from 6 to 24 months of age, and 1/3 of this time was due to the delayed vaccination. Another concern was that province-wide measles endemics had occurred in the last decade and the proportion of cases under 2 years had increased over time in Zhejiang province [4].

The reasons for non-vaccinations of MCV1 and drop-outs of MCV2 included child’s sickness when the vaccination was due, incompatible schedule of the immunization clinic with working hours, and the overcrowded immunization clinic. Drop-outs were also reported in Kenyan [13], where caregivers’ education level and the distance to the health facilities were considered as the important determinants. These findings indicated obstacles to receiving vaccination service still existed despite of the good geographical and financial accessibilities. There were some potential explanations. For example, parents might have misinformation that children with mild sickness could not be vaccinated. Parents received one or more poor immunization services (such as long waiting time) or time constraints might be unwilling to return. Another possible explanation for the low coverage of MCV2 was that caregivers might be less likely to pay attention to MCV2 which scheduled at 18 months of age, because most of the childhood vaccination was scheduled before the first year of life.

Several determinants of the delayed vaccination of MCV1 and MCV2 were identified in this study. First, high numbers of siblings was associated with the delayed MCV vaccinations. These families might face more resource and time constrains to support more children. Resources and parental attentions would be diverted and childhood immunization might not be prioritized amidst competing other demands as the benefits of vaccination might not be apparent immediately [11, 14]. Second, children with hospital delivery were more likely to be timely vaccinated, which was similar to the results in other settings [15, 16]. We inferred that mothers delivered at hospitals might use vaccination services more frequently as they received the better immunization knowledge and awareness conferred by the obstetricians or midwives. Third, children who had younger mothers were more likely to have the delayed vaccination of MCV2. We assumed that older mothers would have more experience in utilization of health care services, which led to an increase in timeliness. Fourth, higher maternal education level was demonstrated a determinant of the timely childhood vaccination. We assumed that a high education level would help mothers get a better understanding and acceptance of vaccination knowledge in practice, through a better communication with vaccination providers [17, 18]. Fifth, mothers with fixed jobs might not have enough time to spend on the childhood immunization or have time constraints due to the inflexible working hours. Sixth, non-local children were more probably to have the delayed MCV vaccinations as their parents might always face the challenge to survive in a new environment with the higher cost of living and might have difficulties in adapting to a new socio-cultural environment. Lastly, we found children with the poorer economic background were probably to have the delayed MCV vaccinations. Previous studies reported that it could be explained as the inaccessibility which was caused by the indirect costs (such as the transport fee) or the deduction of salary for the work leave to bring children to the immunization clinics [3, 16]. There were more determinants for the delayed vaccination of MCV2 than MCV1. We inferred that parents might take more attention on the vaccination scheduled during the first year of life, which might increase the probability of the timeliness of MCV1.

To our knowledge, this study was the first time to evaluate the coverage, the timeliness and completeness of MCV vaccination in Zhejiang province. The reasons for non-vaccination and the determinants of delayed vaccination would help the decision makers to improve the strategies on the routine MCV vaccination. However, there were still several limitations. First, our analyses excluded the children without any written immunization records. It might influence the internal validity of the results within the target population as the vaccination status of these children might differ from those with the immunization records. However, the exclusion reduced the probability of recall bias and only 4 out of 1386 children without any written records. As such, the information bias could be ignored. Second, we could not controlled all confounders for vaccination coverage because of the data limitations. Thus, the impact from other variables like knowledge, attitude on MCV vaccination could not be evaluated. Third, the results of this study would only represent the situation in Zhejiang province and might not be appropriately generalized to other provinces in China.

### Public health recommendations

Based on our findings, we make some recommendations as following: first, sick children should always be screened for vaccination according to the Chinese immunization regulations. Second, the providers should send vaccination reminder (due soon) or recall (past due) through short text messages to reduce the drop-outs [9]. Third, areas with the low coverage of MCV need to develop and implement strategies to reach children who are difficult to reach and monitor the vaccination coverage continuously. Fourth, reaching 95% coverage for MCV1 as early as possible for children aged ≥8 months and reaching 95% coverage for full series of MCV as early as possible for children aged ≥18 months need be emphasized to achieve the goal of measles elimination. Fifth, it is necessary to establish a system to inform mothers on the importance of timely vaccination, aside from using the traditional education methods.

## Conclusions

To summarize, the results of this study demonstrated that the coverage of MCV had improved though one or two cities had not reached the threshold of halting measles transmission. However, the timeliness of MCV still needs improvement. Thus, we suggest the policy-makers pay more attention to the reasons for non-vaccination and determinants of delayed MCV identified in this study when planning efforts to ensure the high age-appropriate coverage of the MCV.

## Abbreviations

CDC: Center of Disease Control and Prevention
CI: Confidence Interval
EPI: Expanded Program on Immunization
HR: Hazard Ratios
MCV: measles-containing vaccine
WHO: World Health Organization
ZJIIS: Zhejiang Provincial Immunization Information System
